# Complete genome sequences of *Enterococcus casseliflavus* strains ASE2 and ASE4 generated by PacBio sequencing

**DOI:** 10.1128/mra.00660-24

**Published:** 2024-09-04

**Authors:** Thao D. Tran, SangIn Lee, Robert Hnasko, Jeffery A. McGarvey

**Affiliations:** 1 U.S. Department of Agriculture, Foodborne Toxin Detection and Prevention Research Unit, Agricultural Research Service, Albany, California, USA; 2 U.S. Department of Agriculture, Produce Safety and Microbiology Research Unit, Agricultural Research Service, Albany, California, USA; The University of Arizona, Tucson, Arizona, USA

**Keywords:** biocontrol, *Enterococcus*, *Salmonella*, *Escherichia coli*

## Abstract

*Enterococcus casseliflavus* strains ASE2 and ASE4 were isolated from the soil of a lettuce farm in Salinas, California, in 2020. Their complete genome sequences were generated by the PacBio Sequel II platform. They both consist of a single circular chromosome without plasmids.

## ANNOUNCEMENT

*Enterococcus casseliflavus* is a motile, facultative Gram-positive bacterium (1, 2). *E. casseliflavus* ASE2 and ASE4 were isolated from agricultural soils from Salinas, CA [as described previously (3)] that were found to be suppressive to the persistence of both *Escherichia coli* O157:H7 and *Salmonella enterica* Poona using the method of Devarajan et al. (4). Briefly, soil between the rows of lettuce was sampled, blended, diluted in phosphate buffer saline, and plated onto Reasoner’s 2A agar plates (for ASE2) or brain heart infusion agar plates (for ASE4) supplemented with cycloheximide (40 mg/L) and incubated at 25°C for 3 days. A single colony was consecutively passaged three times before streaking it onto a tryptic soy agar plate (OXOID, England). The single colony was then grown overnight in tryptic soy broth at 37°C for 24 h with shaking at 200 rpm and harvested for genomic DNA extraction using sucrose-Tris with phenol-chloroform cleanup extractions as described previously (5). Five micrograms of the genomic DNA was sheared using a G-tube (Covaris, MA) at 5,400 × *g* in a MiniSpin plus microcentrifuge (Eppendorf, CT) to target fragment lengths of 7–12 kb. The fragments were not size selected after shearing. The SMRTbell whole-genome libraries (ASE2 and ASE4) were prepared as instructed by the SMRTbell prep kit 3.0 procedure and checklist, barcoded using SMRTbell barcoded adapter plate 3.0 (PacBio, Menlo Park, CA), and sequenced in one single-molecule real-time SMRT cell (120 pM on-plate concentration, Sequel II binding kit 3.2, diffusion loading type, chemistry 11.0, HiFi reads).

The PacBio Sequel II run produced 100,086,616 raw reads in total. The raw data were demultiplexed using Demultiplex Barcodes analysis, run through Circular Consensus Sequencing (CCS) application to obtain a Hifireads file, and assembled using Microbial Genome analysis (SMRT Link v11.1, PacBio, Menlo Park, CA). The CCS analysis result was used to assemble one chromosomal contig and circularized using SMRT Link v11.1 with Microbial Genome Analysis application (PacBio, Menlo Park, CA). No genome rotation was performed. PacBio DNA internal control complex 3.2 was used as an internal sequencing control. The genomes were submitted to the NCBI Prokaryotic Genome Annotation Pipeline v6.4 (PGAP) for annotation (6). Default parameters were used for all software. All characteristics of the ASE2 and ASE4 genome assembly are presented in Table 1.

**TABLE 1:** Characteristics of the genomes of *E. casseliflavus* strains ASE2 and ASE4^*a*^^,^^*b*^

Characteristics	ASE2	ASE4
Genome characteristics		
Genome size (bp)	3,623,851	3,662,325
Coverage (x)	2,571	2,039
GC content (%)	42.7	42.9
Contig(s)	1	1
Circular	Yes	Yes
Coding sequences	3,287	3,360
Coding genes	3,267	3,348
Genome alignment to *E. casseliflavus*	97.9	94.2
ECB140 (% identical sites)^*c*^		
Genome assembly		
Raw reads	36,535,984	28,312,050
CCS analysis HiFi reads	1,246,140	980,251
Median HiFi read quality	Q43	Q43
Mean HiFi read length (bp)	7,511	7,651
N50 mapped CCS read length (bp)	8,065	8,163
Data availability		
GenBank accession	CP119296	CP119393
SRA accession (Hifi reads)	SRS16936858	SRS16943667
BioProject	PRJNA940498	PRJNA940817
BioSample	SAMN33571904	SAMN33579112

^*a*^ CCS, circular consensus sequencing.

^*b*^ SRA, sequence read archive.

^*c*^ Analyzed using LASTZ whole-genome alignment, Geneious Prime 2024.0.3.

*E. casseliflavus* ASE2 and ASE4 genome sequences have been deposited in DDBJ/ENA/GenBank (Table 1). The HiFi reads sets of CCS analysis results for ASE2 and ASE4 are available via sequence read archive (Table 1).
